# The Effects of Ischemia During Rest Intervals on Bar Velocity in the Bench Press Exercise With Different External Loads

**DOI:** 10.3389/fphys.2021.715096

**Published:** 2021-08-10

**Authors:** Jakub Jarosz, Robert Trybulski, Michał Krzysztofik, Athanasios Tsoukos, Aleksandra Filip-Stachnik, Adam Zajac, Gregory C. Bogdanis, Michal Wilk

**Affiliations:** ^1^Institute of Sport Sciences, Jerzy Kukuczka Academy of Physical Education in Katowice, Katowice, Poland; ^2^Department of Medical Sciences, The Wojciech Korfanty School of Economics, Katowice, Poland; ^3^Provita Zory Medical Center, Zory, Poland; ^4^School of Physical Education and Sport Science, National and Kapodistrian University of Athens, Athens, Greece

**Keywords:** blood flow restriction, occlusion, resistance exercise, sport performance, training, high-velocity resistance training, explosive strength

## Abstract

The main aim of the present study was to evaluate the acute effects of ischemia used during rest periods on bar velocity changes during the bench press exercise at progressive loads, from 20 to 90% of 1RM. Ten healthy resistance trained men volunteered for the study (age = 26.3 ± 4.7 years; body mass = 89.8 ± 6.3 kg; bench press 1RM = 142.5 ± 16.9 kg; training experience = 7.8 ± 2.7 years). During the experimental sessions the subjects performed the bench press exercise under two different conditions, in a randomized and counterbalanced order: (a) ischemia condition, with ischemia applied before the first set and during every rest periods between sets, and (b) control condition where no ischemia was applied. During each experimental session eight sets of the bench press exercise were performed, against loads starting from 20 to 90% 1RM, increased progressively by 10% in each subsequent set. A 3-min rest interval between sets was used. For ischemia condition the cuffs was applied 3 min before the first set and during every rest period between sets. Ischemia was released during exercise. The cuff pressure was set to ∼80% of full arterial occlusion pressure. The two-way repeated measures ANOVA showed a statistically significant interaction effect for peak bar velocity (*p* = 0.04) and for mean bar velocity (*p* = 0.01). There was also a statistically significant main effect of condition for peak bar velocity (*p* < 0.01) but not for mean bar velocity (*p* = 0.25). The *post hoc* analysis for interaction showed significantly higher peak bar velocity for the ischemia condition compared to control at a load of 20% 1RM (*p* = 0.007) and at a load of 50% 1RM (*p* = 0.006). The results of the present study indicate that ischemia used before each set even for a brief duration of <3 min, has positive effects on peak bar velocity at light loads, but it is insufficient to induce such effect on higher loads.

## Introduction

Ischemia used in athletic training and rehabilitation is the temporary restriction of blood flow to the arms or legs through external compression (Eltzschig and Eckle, 2011; Schwiete et al., 2021). This compression is usually induced by inflatable cuffs or elastic bands which are wrapped around the proximal parts of the upper or lower limbs (Loenneke et al., 2012). Ischemia induced by a cuff is simple and non-invasive, and therefore easy for practical use (Marocolo et al., 2018). There are many methods of using ischemia as part of physical activity, such as continuous ischemia (used during exercise and rest periods) (Wilk et al., 2020b), intermittent ischemia (used only during exercise) (Wilk et al., 2020d,e), pre-conditioning ischemia (used before exercise) (Telles et al., 2020) and intra-conditioning (used only during the rest periods) (Wilk et al., 2021b). The use of ischemia during exercise, apart from inducing physiological responses, may be accompanied by high ratings of perceived exertion and discomfort or even pain among practitioners (Wernbom et al., 2006; Neto et al., 2018; Schwiete et al., 2021). A way to reduce this discomfort may be to reduce the time during which ischemia is applied, by using it only in the rest intervals between sets of resistance exercise (Yasuda et al., 2015; Freitas et al., 2019, 2020; Wilk et al., 2021b).

Previous studies demonstrated the beneficial effects of ischemic pre-conditioning on performance during different types of exercise (swimming, running, cycling, resistance exercise) (Marocolo et al., 2015, 2016; Ferreira et al., 2016; Paradis-Deschênes et al., 2016; Sabino-Carvalho et al., 2017). Ischemia improves exercise performance and stimulates several physiological responses (Liu et al., 1991; Lawson and Downey, 1993; Pang et al., 1995; Schroeder et al., 1996; Kimura et al., 2007; Li et al., 2012; Paradis-Deschênes et al., 2016; Tanaka et al., 2018), albeit the mechanisms underlying its effects have not been explored exhaustively. Kocman et al. (2015) suggested that the muscles previously subjected to ischemia, become more resistant to ischemia during exercise and its potential deleterious effects. Furthermore, ischemia used before exercise improves metabolic efficiency by attenuating ATP depletion, as well as glycogen depletion and lactate production (Pang et al., 1995; Schroeder et al., 1996; Addison et al., 2003; Lintz et al., 2013). Further, the ischemia pre-conditioning can improve blood flow in skeletal muscles, by inducing vasodilation (Enko et al., 2011), improving functional sympatholysis (Liu et al., 1991), and preserving microvascular endothelium function during stress (Kharbanda et al., 2002; Wang et al., 2004; Bailey et al., 2012; Horiuchi et al., 2015). A recent work by Wilk et al. (2021b) also showed a beneficial effect of ischemic intra-conditioning on explosive performance. In that study there was an increase in bar velocity and power output during the bench press exercise (five sets, three repetitions, 60% of one repetition maximum-RM; 5 min rest interval) when ischemia 80% of arterial occlusion pressure-AOP was used before the first set and during all rest periods between sets. Similarly, beneficial effects on explosive bench press performance were observed when ischemia was applied during the exercise (Wilk et al., 2020b,c). However, the positive effects of different types of ischemia during exercise (intermittent and continuous at 70% AOP) seem to be depended on external load used (Wilk et al., 2020b). Specifically, the positive effects on peak bar velocity during the bench press exercise were evident in lighter loads (20–50% 1RM) but disappeared when heavier loads (60–90% 1RM) were used (Wilk et al., 2020b). On the contrary, Wilk et al. (2020e) showed that intermittent, high pressure ischemia (90% AOP) significantly increased power output and bar velocity during the bench press exercise at a load of 70% 1RM, but only when a wider cuff was used, while there was no effect when a narrow cuff was used. Therefore, the benefits of ischemia on exercise performance appear to depend on the external load used, as well as the width and pressure of the cuff. However, the relationship between the external load used and the effects of ischemia applied during the rest intervals only, has not yet been examined. Therefore, it seems justified to determine whether there is a relationship between the changes in bar velocity and the external load used, when ischemia is applied only during the rest intervals between sets.

The bench press exercise was selected, as it is the most popular upper-body resistance exercise, commonly used in practice and science research (Wilk et al., 2019c; Maszczyk et al., 2020; Filip-Stachnik et al., 2021b; Krzysztofik et al., 2021), while ischemia during the rest intervals only may be more tolerated than continuous ischemia during resistance training. Thus, the main aim of the present study was to evaluate the acute effects of ischemia used during rest periods on bar velocity changes during the bench press exercise at progressive loads, from 20 to 90% of 1RM. Since previous studies showed a beneficial effect of resting ischemia on physical performance (Telles et al., 2020), it was hypothesized that ischemia used only during the rest intervals would increase bar velocity during the bench press exercise at all loads used.

## Materials and Methods

All stages of the study were performed in the Strength and Power Laboratory at the Academy of Physical Education in Katowice, Poland. A randomized crossover design was used, where each participant performed two training protocols in a random and counterbalanced order, 1 week apart: with ischemia used before exercise and during the rest intervals between sets (ischemia condition), and a control condition, without ischemia. During each training session, the participants performed eight sets of the bench press exercise with two repetitions in each set, with progressive loads from 20 to 90% 1RM (10% steps), and 3 min rest periods between each set. During the ischemia condition occlusion with 80% AOP was applied using a 10 cm wide cuff, before the first set of the bench press exercise and during all rest periods between sets. The ischemia was applied in close proximity to the axillary’s fossa of both arms. During the control condition no ischemia was applied.

### Subjects

Power analysis indicated a sample size of eight participants would be needed to detect significant differences if the effect size (ES) was 0.25. Power analysis was performed using the following parameters: type of analysis was set to repeated-measures ANOVA (within factors), the required power (1-β error) was set to 0.80, alpha was set to 0.05, and the correlation coefficient among repeated measures was set to 0.5 (G-Power software, v.3.1.9.2).

Ten healthy resistance trained men volunteered for the study [age = 26.3 ± 4.7 years; body mass = 89.8 ± 6.3 kg; bench press 1RM = 142.5 ± 16.9 kg; training experience = 7.8 ± 2.7 years; relative strength (1RM/body mass) = 1.59 ± 0.13]. The inclusion criteria were a bench press 1RM performance of at least 150% body mass (Wilk et al., 2019b) and no musculoskeletal injuries for at least 6 months prior to the study. Participants were instructed to maintain their normal dietary habits over the course of the study and not to use any supplements or stimulants for the duration of the experiment. The participants were informed about the potential risks of the study before providing their written informed consent for participation and were allowed to withdraw from the study at any time. The study protocol was approved by the Bioethics Committee for Scientific Research, at the Academy of Physical Education in Katowice, Poland (02/2019), and all procedures were in accordance with the ethical standards of the Declaration of Helsinki, 1983.

### Procedures

#### Familiarization Session

Two weeks before the main experiment, the participants performed a familiarization session. Familiarization, and experimental sessions were performed at the same time of the day, between 9:00 a.m. and 11:00 a.m., to avoid the influence of circadian rhythm on performance. During the familiarization session, the participants performed a warm-up that was consistent with subjects normal training habits, followed by a specific warm-up during which they performed the free-weight barbell bench press exercise at 20 kg and then at 40%, 60% of their estimated 1RM with 8, 6, and 3 repetitions, respectively. During each warm-up set the participants performed 5–8 repetitions. During the familiarization sessions each participant performed six sets of the bench press exercise with ischemia used during the rest intervals. The load was increased by 10% from 40 to 80% of the estimated 1RM. In each set, two repetitions with maximal movement tempo were performed.

#### One Repetition Maximum Test (1RM)

One week before the main experiment a 1RM free-weight barbell bench press test was performed as described elsewhere (Filip-Stachnik et al., 2021a; Wilk et al., 2021a). The 1RM test is considered the gold standard for assessing muscle strength under non-laboratory conditions (Levinger et al., 2009). Briefly, the session started with the general upper body warm-up, similar to that performed during familiarization. Afterwards, the participants performed specific bench press warm-up at a load of 20, 40, and 60% of their estimated 1RM with 8, 6, and 3 repetitions, respectively. The first testing load was set to an estimated 80% 1RM and was increased by 2.5–10 kg for each subsequent trial. This process was repeated until failure. Grip width on the bar was set at 150% of the individual bi-acromial distance, and the same grip width was used in the main experimental sessions (Wilk et al., 2019a).

#### Experimental Sessions

The experimental procedure was similar to that described in the study of Wilk et al. (2020b) with the exception of the ischemia method. The subjects performed the free-weight barbell bench press exercise under two different conditions, in a randomized and counterbalanced order: (a) ischemia condition, with ischemia applied before the first set and during every rest interval between sets, and (b) control condition where no ischemia was applied. During each experimental session eight sets of the bench press exercise were performed, against loads starting from 20 to 90% 1RM, increased progressively by 10% in each subsequent set. In each set, two repetitions were performed. Research has shown that the fastest movement velocity (peak and mean) is achieved in the first two repetitions (García-Ramos et al., 2021). Furthermore it was chosen two repetitions to avoid the cumulative fatigue in the last loads. Each repetition was performed with a 2 s duration for the eccentric phase and maximal velocity in the concentric phase of movement (Wilk et al., 2020a,c). A 3-min rest interval between sets was used. Bar velocity was monitored using a linear position transducer system (Tendo Power Analyzer, Tendo Sport Machines, Trencin, Slovakia). This device has shown high reliability and validity, intra-class correlation co-efficient (ICC) of 0.984 for peak bar velocity and 0.989 for mean bar velocity, and a coefficient of variation (CV) ranging from 9 to 9.6% (Garnacho-Castaño et al., 2015). Peak bar velocity was obtained from the best repetition performed in each set. Mean bar velocity was obtained as the mean of two repetitions performed in each set.

### Ischemia

Ischemia was induced by cuffs worn at the most proximal region of both arms. For this experiment we used SmartCuffs (Smart Tools Plus LLC, Strongsville, OH, United States) which are 10-cm wide. The ischemia was applied 3 min before the first set and during every rest period between sets. The ischemia was applied for approximately 2.5 min during each rest interval, as it took about 20 s to inflate and 10 s to deflate the cuffs. Occlusion was released during exercise. The cuff pressure was set to ∼80% of full arterial occlusion pressure (115 ± 10 mmHg). The individual pressure value was determined by a handheld Edan SD3 Doppler with an OLED screen and a 2-mHz probe (Edan Instruments Inc., Shenzhen, China) placed over the radial artery to assess the blood pressure required for interruption of auscultatory pulse (Wilk et al., 2020b,e, 2021b).

#### Statistical Analysis

All statistical analyses were performed using Statistica 9.1. Results are presented as means with standard deviations. The Shapiro–Wilk, Levene, and Mauchly’s tests were used to verify normality, homogeneity, and sphericity of the sample data variances, respectively. Differences between the ischemia and control conditions were examined using two-way repeated measures ANOVA [2 conditions (ischemia vs. control) × 8 sets (load 20–90% 1RM)]. ES for main effects and interactions were determined by partial eta squared (η^2^). Partial eta squared values were classified as small (0.01–0.059), moderate (0.06–0.137), and large (>0.137) (Hopkins et al., 2009). *Post hoc* comparisons using the Tukey’s test were conducted to locate the differences between mean values, when a main effect or an interaction was found. For pairwise comparisons, ESs were determined by Cohen’s *d* which was characterized as large (*d* > 0.8), moderate (*d* between 0.8 and 0.5), small (*d* between 0.49 and 0.20), and trivial (*d* < 0.2) (Cohen, 1988). Percent changes with 90% confidence intervals (90CI) were also calculated. Statistical significance was set at *p* < 0.05.

## Results

Peak and mean bar velocity in all sets of the two conditions are presented in Table 1. The two-way repeated measures ANOVA showed a statistically significant interaction effect for peak bar velocity (*p* = 0.04; η^2^ = 0.20) and for mean bar velocity (*p* = 0.01; η^2^ = 0.20). There was also a statistically significant main effect of condition for peak bar velocity (*p* < 0.01; η^2^ = 0.40) but not for mean bar velocity (*p* = 0.25; η^2^ = 0.15).

**TABLE 1:** Bar velocity during eight sets of the bench press exercise under two conditions.

Condition	20% 1RM	30% 1RM	40% 1RM	50% 1RM	60% 1RM	70% 1RM	80% 1RM	90% 1RM
**Peak bar velocity (m/s)**								
Control	2.17 ± 0.24 (2.04–2.31)	1.81 ± 0.16 (1.72–1.90)	1.52 ± 0.32 (1.33–1.71)	1.21 ± 0.25 (1.06–1.35)	1.04 ± 0.15 (0.96–1.13)	0.90 ± 0.11 (0.83–0.96)	0.72 ± 0.11 (0.66–0.78)	0.56 ± 0.13 (0.48–0.63)
Ischemia	2.30 ± 0.24 (2.16–2.44)	1.90 ± 0.24 (1.76–2.04)	1.58 ± 0.29 (1.41–1.75)	1.34 ± 0.21 (1.21–1.46)	1.10 ± 0.17 (1.00–1.20)	0.93 ± 0.15 (0.84–1.02)	0.72 ± 0.16 (0.63–0.81)	0.59 ± 0.15 (0.5–0.68)
Control vs. Ischemia (*p*-value)	0.007*	0.264	0.836	0.006*	0.852	0.998	1.000	0.997
Cohen’s *d*	0.56	0.45	0.20	0.58	0.38	0.28	0.01	0.27
**Mean bar velocity (m/s)**								
Control	1.44 ± 0.16 (1.39–1.60)	1.24 ± 0.11 (1.15–1.35)	1.03 ± 0.20 (0.95–1.17)	0.86 ± 0.13 (0.81–1.01)	0.74 ± 0.13 (0.66–0.82)	0.62 ± 0.11 (0.57–0.70)	0.50 ± 0.10 (0.43–0.55)	0.37 ± 0.10 (0.28–0.43)
Ischemia	1.50 ± 0.18 (1.34–1.53)	1.25 ± 0.18 (1.18–1.31)	1.06 ± 0.19 (0.91–1.15)	0.91 ± 0.17 (0.78–0.94)	0.74 ± 0.14 (0.66–0.82)	0.64 ± 0.11 (0.55–0.68)	0.49 ± 0.11 (0.44–0.56)	0.36 ± 0.13 (0.31–0.43)
Control vs. Ischemia (*p*-value)	0.088	1.000	0.996	0.372	1.000	0.999	0.999	0.999
Cohen’s *d*	0.40	0.06	0.13	0.36	0.02	0.21	0.13	0.15

Values are mean ± SD, while 90% confidence intervals (CI) are presented in parentheses. *p < 0.01 from the corresponding value in the control condition.

The *post hoc* analysis for interaction showed significantly higher peak bar velocity for the ischemia condition compared to control at a load of 20% 1RM (2.30 ± 0.24 vs. 2.17 ± 0.24 m/s, *p* = 0.007) and at a load of 50% 1RM (1.34 ± 0.21 vs. 1.21 ± 0.25 m/s, *p* = 0.006, Table 1).

## Discussion

The main finding of the present study was that ischemia used before each set significantly increased peak bar velocity during the bench press exercise only against the 20 and 50% 1RM loads. Furthermore, no positive effects of ischemia were observed on mean bar velocity. Therefore, it appears that the effect of ischemia on peak bench press performance was limited to lighter load, while increasing the load during the subsequent sets canceled out any effects of between-sets ischemia on performance.

Previous studies have confirmed that muscular ischemia during exercise (Gepfert et al., 2020) or before exercise increases physical performance in various types of physical activity (Telles et al., 2020). However, only one previous study assessed the effect of ischemia applied before exercise and during rest intervals between sets of resistance exercise on explosive performance. In that study, ischemia significantly increased peak power output and peak bar velocity during the bench press exercise, while it had no effect on mean values (Wilk et al., 2021b). However, in the present study, the bench press exercise was performed against increasing loads (8 sets at 20–90% 1RM), while in the study of Wilk et al. (2021b) the load was constant for all sets (5 sets at 60% 1RM). This indicates that the increase in load during subsequent sets cancels any positive effects of between-sets ischemic on performance. Thus, ischemia applied before the first sets, appears to be beneficial for peak bench press performance at 20 and 50% 1RM (Table 1). Similar findings regarding the positive effect of ischemic preconditioning have been observed during strength-endurance resistance exercise (Marocolo et al., 2015). In that study, the number of repetitions during leg extension exercise to exhaustion was increased following ischemia compared to control (Marocolo et al., 2015). Furthermore, ischemia used during rest periods may enhance ATP production by glycolytic and phosphogenic pathways (Janier et al., 1994; Mendez-Villanueva et al., 2012), as well as peak contractile force (Andreas et al., 2011). Since the level of power output generated by the muscle depends on these substrates and metabolic mechanisms (Kraemer et al., 1987; Robergs et al., 1991), this may be the physiological basis for explaining the obtained results. Furthermore, the reactive hyperemia (during the reperfusion phase after occlusion) may be associated with potentiated force production and with a beneficial effect on explosive performance (Libonati et al., 1998; Marocolo et al., 2015).

Although that present study showed a main effect of condition in peak bar velocity, which favored the ischemia condition, this was due mainly to the difference in the light load, as shown by the *post hoc* test following the significant load × condition interaction. Comparisons between corresponding sets of ischemia and control in peak bar velocity showed statistically significant differences with small to moderate ES at least up to a load of 50% 1RM (ES = 0.20–0.58, Table 1). Therefore, there are some other possible factors that may explain the tendency of ischemia during recovery to increase peak bar velocity. One such factor may be the duration of ischemia. In the study Wilk et al. (2021b) the ischemia was applied for approximately 5 min during each rest period, while in the present study the rest interval was approximately half (i.e., 2.5 min). Previous studies have examined the effect of ischemia pre-conditioning lasting from 6 to 20 min (Lalonde and Curnier, 2015). The most common ischemia protocol involves three or four cycles of 5 min of ischemia and reperfusion (Murry et al., 1986; de Groot et al., 2010; Jean-St-Michel et al., 2011). Therefore, the duration of ischemia used in this study could be insufficient to induce improvement of explosive performance at higher loads, and this was also observed in the study by Wilk et al. (2021b). Nevertheless, it should be emphasized that the optimal methodology for implementing ischemia is unknown (Sharma et al., 2014; Incognito et al., 2016). The characteristics of the ischemia protocols, such as the pressure applied, training experience, type and intensity of exercise used, number of ischemia–reperfusion cycles, and time between the removal of ischemia until the start of exercise, are certainly factors that influence the effectiveness of ischemia application (Incognito et al., 2016; Wilk et al., 2018). However, currently there are no clear guidelines for the optimal methodology of ischemia application according to individual characteristics and training variables.

Furthermore, the ischemic conditioning may delay the development of fatigue and prolong the time to task failure, as demonstrated by Barbosa et al. (2015) during grip exercises, however, the reported improvement was not accompanied by physiological changes (e.g., increased blood flow or oxygen utilization). Similar Marocolo et al. (2015) showed that ischemia preconditioning (four cycles of 5 min of occlusion at 220 mmHg) increased the number of repetitions during leg extension exercise. However, the same improvement as for ischemia condition was noted for the placebo condition (20 mmHg). Therefore, there may be additional, unknown factors that influence the effect of ischemia on exercise performance. One limitation of the present study was the lack of a placebo condition. The placebo and/or nocebo effects are both methodological confounding factors in studies involving potential ergogenic aids (Ferreira et al., 2016; Marocolo et al., 2017; Wilk et al., 2019c). Another limitation of the present study is the lack of assessment of physiological changes that could constitute the basis for explaining the obtained results. Since this study is the second one that evaluates the acute effects of ischemia used during the rest periods between sets, further research on such ischemia application practices are required. Furthermore, in presented study the successive sets were performed using loads in an ascending order. Although an order effect of load is possible, the effect of ischemia on performance was observed in the initial sets, i.e., first (20% 1RM) and fourth set (50% 1RM), while no difference was seen in the last sets. Nevertheless, despite the low number of repetitions per set (only two) and the relatively long rest interval between sets (3 min), it is possible that fatigue may have affected performance in the last sets, thus confounding a possible positive effect of ischemia. Therefore, further research is needed, examining the effects of load on ischemia-induced performance enhancement using randomized loads.

## Conclusion

Ischemia application during and between exercise bouts is an innovative intervention allowing to temporarily increase exercise capacity and efficiency (Incognito et al., 2016; Marocolo et al., 2017). The results of the present study indicate that ischemia used before each set even for a brief duration of <3 min, has positive effects on peak bar velocity at light loads (20–50%), but it is insufficient to induce such effect on higher loads. However, in subsequent sets, the effect of increasing load is stronger than that of ischemia during the recovery interval, thus preventing any further increase in explosive performance. Although more research is needed to determine the effects of ischemia application during the recovery intervals between sets of exercise, it seems that the duration of ischemia application during recovery (i.e., <3 min) was not sufficient to induce positive changes in performance.

## Data Availability Statement

The original contributions presented in the study are included in the article/Supplementary Materials, further inquiries can be directed to the corresponding author/s.

## Ethics Statement

The studies involving human participants were reviewed and approved by The Bioethics Committee for Scientific Research, at the Academy of Physical Education in Katowice, Poland (02/2019). The patients/participants provided their written informed consent to participate in this study.

## Author Contributions

JJ, RT, and MW: study conception and design. JJ and AF-S: acquisition of data. MW, AT, and GB: analysis and interpretation of data. MW, MK, RT, and AF-S: drafting of manuscript. MW, AZ, AT, and GB: critical revision. All authors contributed to the article and approved the submitted version.

## Conflict of Interest

The authors declare that the research was conducted in the absence of any commercial or financial relationships that could be construed as a potential conflict of interest.

## Publisher’s Note

All claims expressed in this article are solely those of the authors and do not necessarily represent those of their affiliated organizations, or those of the publisher, the editors and the reviewers. Any product that may be evaluated in this article, or claim that may be made by its manufacturer, is not guaranteed or endorsed by the publisher.

## References

[B1] Addison P. D. Neligan P. C. Ashrafpour H. Khan A. Zhong A. Moses M. (2003). Noninvasive remote ischemic preconditioning for global protection of skeletal muscle against infarction. *Am. J. Physiol. Heart Circ. Physiol.* 285 H1435–H1443. 10.1152/ajpheart.00106.2003 12791590

[B2] Andreas M. Schmid A. I. Keilani M. Doberer D. Bartko J. Crevenna R. (2011). Effect of ischemic preconditioning in skeletal muscle measured by functional magnetic resonance imaging and spectroscopy: a randomized crossover trial. *J. Cardiovasc. Magn. Reson.* 13:32. 10.1186/1532-429X-13-32 21718491 PMC3143996

[B3] Bailey T. G. Birk G. K. Cable N. T. Atkinson G. Green D. J. Jones H. (2012). Remote ischemic preconditioning prevents reduction in brachial artery flow-mediated dilation after strenuous exercise. *Am. J. Physiol. Heart Circ. Physiol.* 303 H533–H538. 10.1152/ajpheart.00272.2012 22730390

[B4] Barbosa T. C. Machado A. C. Braz I. D. Fernandes I. A. Vianna L. C. Nobrega A. C. (2015). Remote ischemic preconditioning delays fatigue development during handgrip exercise. *Scand. J. Med. Sci. Sports* 25 356–364. 10.1111/sms.12229 24731023

[B5] Cohen J. (1988). *Statistical Power Analysis for the Behavioral Sciences, 2nd Edn.* Hillsdale, NJ: L. Erlbaum Associates.

[B6] de Groot P. C. Thijssen D. H. Sanchez M. Ellenkamp R. Hopman M. T. (2010). Ischemic preconditioning improves maximal performance in humans. *Eur. J. Appl. Physiol.* 108 141–146. 10.1007/s00421-009-1195-2 19760432 PMC2793394

[B7] Eltzschig H. K. Eckle T. (2011). Ischemia and reperfusion–from mechanism to translation. *Nat. Med.* 17 1391–1401. 10.1038/nm.2507 22064429 PMC3886192

[B8] Enko K. Nakamura K. Yunoki K. Miyoshi T. Akagi S. Yoshida M. (2011). Intermittent arm ischemia induces vasodilatation of the contralateral upper limb. *J. Physiol. Sci.* 61 507–513. 10.1007/s12576-011-0172-9 21901641 PMC10718035

[B9] Ferreira T. N. Sabino-Carvalho J. L. Lopes T. R. Ribeiro I. C. Succi J. E. Da Silva A. C. (2016). Ischemic preconditioning and repeated sprint swimming: a placebo and nocebo study. *Med. Sci. Sports Exerc.* 48 1967–1975. 10.1249/MSS.0000000000000977 27187105

[B10] Filip-Stachnik A. Krzysztofik M. Kaszuba M. Leznicka K. Kostrzewa M. Del Coso J. (2021a). Effects of acute caffeine intake on power output and movement velocity during a multiple-set bench press exercise among mild caffeine users. *J. Hum. Kinet.* 78 219–228. 10.2478/hukin-2021-0044 34025879 PMC8120957

[B11] Filip-Stachnik A. Wilk M. Krzysztofik M. Lulińska E. Tufano J. J. Zajac A. (2021b). The effects of different doses of caffeine on maximal strength and strength-endurance in women habituated to caffeine. *J. Int. Soc. Sports Nutr.* 18:25. 10.1186/s12970-021-00421-9 33781269 PMC8008648

[B12] Freitas E. D. S. Miller R. M. Heishman A. D. Aniceto R. R. Silva J. G. C. Bemben M. G. (2019). Perceptual responses to continuous versus intermittent blood flow restriction exercise: a randomized controlled trial. *Physiol. Behav.* 212:112717. 10.1016/j.physbeh.2019.112717 31629764

[B13] Freitas E. D. S. Miller R. M. Heishman A. D. Ferreira-Júnior J. B. Araújo J. P. Bemben M. G. (2020). Acute physiological responses to resistance exercise with continuous versus intermittent blood flow restriction: a randomized controlled trial. *Front. Physiol.* 11:132. 10.3389/fphys.2020.00132 32256374 PMC7090220

[B14] García-Ramos A. Weakley J. Janicijevic D. Jukic I. (2021). Number of repetitions performed before and after reaching velocity loss thresholds: First repetition versus fastest repetition-mean velocity versus peak velocity. *Int. J. Sports Physiol. Perform.* 16, 950–957. 10.1123/ijspp.2020-0629 33691279

[B15] Garnacho-Castaño M. V. Lo’pez-Lastra S. Mate’-Muñoz J. L. (2015). Reliability and validity assessment of a linear position transducer. *J. Sports Sci. Med.* 14 128–136. 25729300 PMC4306764

[B16] Gepfert M. Golas A. Zajac T. Krzysztofik M. (2020). The use of different modes of post-activation potentiation (PAP) for enhancing speed of the slide-step in basketball players. *Int. J. Environ. Res.* 17:5057. 10.3390/ijerph17145057 32674351 PMC7400334

[B17] Hopkins W. G. Marshall S. W. Batterham A. M. Hanin J. (2009). Progressive statistics for studies in sports medicine and exercise science. *Med. Sci. Sports Exerc.* 41 3–12. 10.1249/mss.0b013e31818cb278 19092709

[B18] Horiuchi M. Endo J. Thijssen D. H. (2015). Impact of ischemic preconditioning on functional sympatholysis during handgrip exercise in humans. *Physiol. Rep.* 3:e12304. 10.14814/phy2.12304 25713329 PMC4393211

[B19] Incognito A. V. Burr J. F. Millar P. J. (2016). The effects of ischemic preconditioning on human exercise performance. *Sports Med.* 46 531–544. 10.1007/s40279-015-0433-5 26645697

[B20] Janier M. F. Vanoverschelde J. L. Bergmann S. R. (1994). Ischemic preconditioning stimulates anaerobic glycolysis in the isolated rabbit heart. *Am. J. Physiol.* 267 H1353–H1360. 10.1152/ajpheart.1994.267.4.H1353 7943381

[B21] Jean-St-Michel E. Manlhiot C. Li J. Tropak M. Michelsen M. M. Schmidt M. R. (2011). Remote preconditioning improves maximal performance in highly trained athletes. *Med. Sci. Sports Exerc.* 43 1280–1286. 10.1249/MSS.0b013e318206845d 21131871

[B22] Kharbanda R. K. Mortensen U. M. White P. A. Kristiansen S. B. Schmidt M. R. Hoschtitzky J. A. (2002). Transient limb ischemia induces remote ischemic preconditioning in vivo. *Circulation* 106 2881–2883. 10.1161/01.cir.0000043806.51912.9b 12460865

[B23] Kimura M. Ueda K. Goto C. Jitsuiki D. Nishioka K. Umemura T. (2007). Repetition of ischemic preconditioning augments endothelium-dependent vasodilation in humans: role of endothelium-derived nitric oxide and endothelial progenitor cells. *Arterioscler. Thromb. Vasc. Biol.* 27 1403–1410. 10.1161/ATVBAHA.107.143578 17446439

[B24] Kocman E. A. Ozatik O. Sahin A. Guney T. Kose A. A. Dag I. (2015). Effects of ischemic preconditioning protocols on skeletal muscle ischemia-reperfusion injury. *J. Surg. Res.* 193 942–952. 10.1016/j.jss.2014.09.032 25438960

[B25] Kraemer W. J. Noble B. J. Clark M. J. Culver B. W. (1987). Physiologic responses to heavy-resistance exercise with very short rest periods. *Int. J. Sports Med.* 8 247–252. 10.1055/s-2008-1025663 3667019

[B26] Krzysztofik M. Jarosz J. Matykiewicz P. Wilk M. Bialas M. Zajac A. (2021). A comparison of muscle activity of the dominant and non-dominant side of the body during low versus high loaded bench press exercise performed to muscular failure. *J. Electromyogr. Kinesiol.* 56:102513. 10.1016/j.jelekin.2020.102513 33422743

[B27] Lalonde F. Curnier D. Y. (2015). Can anaerobic performance be improved by remote ischemic preconditioning? *J. Strength Cond. Res.* 29 80–85. 10.1519/JSC.0000000000000609 25068802

[B28] Lawson C. Downey J. (1993). Preconditioning: state of the art myocardial protection. *Cardiovasc. Res.* 27 542–550. 10.1093/cvr/27.4.542 8324783

[B29] Levinger I. Goodman C. Hare D. L. Jerums G. Toia D. Selig S. (2009). The reliability of the 1RM strength test for untrained middle- aged individuals. *J. Sci. Med. Sport* 12 310–316. 10.1016/j.jsams.2007.10.007 18078784

[B30] Li X. D. Cheng Y. T. Yang Y. J. Meng X. M. Zhao J. L. Zhang H. T. (2012). PKA-mediated eNOS phosphorylation in the protection of ischemic preconditioning against no-reflow. *Microvasc. Res.* 84 44–54. 10.1016/j.mvr.2012.04.002 22542438

[B31] Libonati J. R. Cox M. Incanno N. Melville S. K. Musante F. C. Glassberg H. L. (1998). Brief periods of occlusion and reperfusion increase skeletal muscle force output in humans. *Cardiologia* 43 1355–1360. 9988944

[B32] Lintz J. A. Dalio M. B. Joviliano E. E. Piccinato C. E. (2013). Ischemic pre and postconditioning in skeletal muscle injury produced by ischemia and reperfusion in rats. *Acta Circ. Bras.* 28 441–446. 10.1590/s0102-86502013000600007 23743682

[B33] Liu G. S. Thornton J. Van Winkle D. M. Stanley A. W. Olsson R. A. Downey J. M. (1991). Protection against infarction afforded by preconditioning is mediated by A1 adenosine receptors in rabbit heart. *Circulation* 84 350–356. 10.1161/01.cir.84.1.350 2060105

[B34] Loenneke J. P. Fahs C. A. Rossow L. M. Sherk V. D. Thiebaud R. S. Abe T. (2012). Effects of cuff width on arterial occlusion: implications for blood flow restricted exercise. *Eur. J. Appl. Physiol.* 112 2903–2912. 10.1007/s00421-011-2266-8 22143843 PMC4133131

[B35] Marocolo I. C. Da Mota G. R. Londe A. M. Patterson S. D. Barbosa Neto O. Marocolo M. (2017). Acute ischemic preconditioning does not influence high-intensity intermittent exercise performance. *PeerJ* 5:e4118. 10.7717/peerj.4118 29204325 PMC5712465

[B36] Marocolo M. Billaut F. da Mota G. R. (2018). Ischemic preconditioning and exercise performance: an ergogenic aid for whom? *Front. Physiol.* 9:1874. 10.3389/fphys.2018.01874 30622484 PMC6308393

[B37] Marocolo M. Da Mota G. R. Pelegrini V. Appell Coriolano H. J. (2015). Are the beneficial effects of ischemic preconditioning on performance partly a placebo effect? *Int. J. Sports Med.* 36 822–825. 10.1055/s-0035-1549857 26058479

[B38] Marocolo M. Marocolo I. C. da Mota G. R. Simão R. Maior A. S. Coriolano H. J. (2016). Beneficial effects of ischemic preconditioning in resistance exercise fade over time. *Int. J. Sports Med.* 37 819–824. 10.1055/s-0042-109066 27348720

[B39] Maszczyk A. Wilk M. Krzysztofik M. Gepfert M. Zaja̧c A. Petr M. (2020). The effects of resistance training experience on movement characteristics in the bench press exercise. *Biol. Sport.* 37 79–83. 10.5114/biolsport.2019.83008 32205913 PMC7075231

[B40] Mendez-Villanueva A. Edge J. Suriano R. Hamer P. Bishop D. (2012). The recovery of repeated-sprint exercise is associated with PCr resynthesis, while muscle pH and EMG amplitude remain depressed. *PLoS One* 7:e51977. 10.1371/journal.pone.0051977 23284836 PMC3524088

[B41] Murry C. E. Jennings R. B. Reimer K. A. (1986). Preconditioning with ischemia: a delay of lethal cell injury in ischemic myocardium. *Circulation* 74 1124–1136. 10.1161/01.cir.74.5.1124 3769170

[B42] Neto G. R. Novaes J. S. Salerno V. P. Gonçalves M. M. Batista G. R. Cirilo-Sousa M. S. (2018). Does a resistance exercise session with continuous or intermittent blood flow restriction promote muscle damage and increase oxidative stress? *J. Sports Sci.* 36 104–110. 10.1080/02640414.2017.1283430 28143367

[B43] Pang C. Y. Yang R. Z. Zhong A. Xu N. Boyd B. Forrest C. R. (1995). Acute ischaemic preconditioning protects against skeletal muscle infarction in the pig. *Cardiovasc. Res.* 29 782–788. 10.1016/0008-6363(96)88613-5 7656281

[B44] Paradis-Deschênes P. Joanisse D. R. Billaut F. (2016). Ischemic preconditioning increases muscle perfusion, oxygen uptake, and forcein strength-trained athletes. *Appl. Physiol. Nutr. Metab.* 41 938–944. 10.1139/apnm-2015-0561 27574913

[B45] Robergs R. A. Pearson D. R. Costill D. L. Fink W. J. Pascoe D. D. Benedict M. A. (1991). Muscle glycogenolysis during differing intensities of weight-resistance exercise. *J. Appl. Physiol.* 70 1700–1706. 10.1152/jappl.1991.70.4.1700 2055849

[B46] Sabino-Carvalho J. L. Lopes T. R. Obeid-Freitas T. Ferreira T. N. Succi J. E. Silva A. C. (2017). Effect of ischemic preconditioning on endurance performance does not surpass placebo med. *Sci. Sports Exerc.* 49 124–132. 10.1249/MSS.0000000000001088 27580156

[B47] Schroeder C. A. Lee H. T. Shah P. M. Babu S. C. Thompson C. I. Belloni F. L. (1996). Preconditioning with ischemia or adenosine protects skeletal muscle from ischemic tissue reperfusion injury. *J. Surg. Res.* 63 29–34. 10.1006/jsre.1996.0217 8661167

[B48] Schwiete C. Franz A. Roth C. Behringer M. (2021). Effects of resting vs. continuous blood-flow restriction-training on strength, fatigue resistance, muscle thickness, and perceived discomfort. *Front. Physiol.* 12:663665. 10.3389/fphys.2021.663665 33859576 PMC8042206

[B49] Sharma V. Cunniffe B. Verma A. P. Cardinale M. Yellon D. (2014). Characterization of acute ischemia-related physiological responses associated with remote ischemic preconditioning: a randomized controlled, crossover human study. *Physiol. Rep.* 2:e12200. 10.14814/phy2.12200 25413320 PMC4255807

[B50] Tanaka D. Suga T. Tanaka T. Kido K. Honjo T. Fujita S. (2018). Ischemic preconditioning enhances muscle endurance during sustained isometric exercise. *Int. J. Sports Med.* 37 614–618. 10.1055/s-0035-1565141 27176889

[B51] Telles L. G. S. Carelli L. C. Bráz I. D. Junqueira C. Monteiro E. R. Reis V. M. (2020). Effects of ischemic preconditioning as a warm-up on leg press and bench press performance. *J. Hum. Kinet.* 75 267–277. 10.2478/hukin-2020-0055 33312313 PMC7706675

[B52] Wang W. Z. Stepheson L. L. Fang X. H. Khiabani K. T. Zamboni W. A. (2004). Ischemic preconditioning-induced microvascular protection at a distance. *J. Reconstr. Microsurg.* 20 175–181. 10.1055/s-2004-820775 15011127

[B53] Wernbom M. Augustsson J. Thomee R. (2006). Effects of vascular occlusion on muscular endurance in dynamic knee extension exercise at different submaximal loads. *J. Strength Cond. Res.* 20 372–377. 10.1519/R-16884.1 16686566

[B54] Wilk M. Gepfert M. Krzysztofik M. Golas A. Mostowik A. Maszczyk A. (2019a). The influence of grip width on training volume during the bench press with different movement tempos. *J. Hum. Kinet.* 68 49–57. 10.2478/hukin-2019-0055 31531132 PMC6724586

[B55] Wilk M. Gepfert M. Krzysztofik M. Mostowik A. Filip A. Hajduk G. (2020a). Impact of duration of eccentric movement in the one-repetition maximum test result in the bench press among women. *J. Sports Sci. Med.* 19 317–322. 32390725 PMC7196738

[B56] Wilk M. Gepfert M. Krzysztofik M. Stastny P. Zajac A. Bogdanis G. C. (2020b). Acute effects of continuous and intermittent blood flow restriction on movement velocity during bench press exercise against different loads. *Front. Physiol.* 11:569915. 10.3389/fphys.2020.569915 33329020 PMC7728989

[B57] Wilk M. Golas A. Krzysztofik M. Nawrocka M. Zajac A. (2019b). The effects of eccentric cadence on power and velocity of the bar during the concentric phase of the bench press movement. *J. Sports Sci. Med.* 18 191–197. 31191087 PMC6543996

[B58] Wilk M. Golas A. Zmijewski P. Krzysztofik M. Filip A. Coso J. D. (2020c). The effects of the movement tempo on the one-repetition maximum bench press results. *J. Hum. Kinet.* 72 151–159. 10.2478/hukin-2020-0001 32269656 PMC7126254

[B59] Wilk M. Jarosz J. Krzysztofik M. Filip-Stachnik A. Bialas M. Rzeszutko-Belzowska A. (2021a). Contrast tempo of movement and its effect on power output and bar velocity during resistance exercise. *Front. Physiol.* 11:629199. 10.3389/fphys.2020.629199 33551848 PMC7854892

[B60] Wilk M. Krzysztofik M. Filip A. Lockie R. G. Zajac A. (2020d). The acute effects of external compression with blood flow restriction on maximal strength and strength-endurance performance of the upper limbs. *Front. Physiol.* 11:567. 10.3389/fphys.2020.00567 32587525 PMC7298135

[B61] Wilk M. Krzysztofik M. Filip A. Zajac A. Bogdanis G. C. Lockie R. G. (2020e). Short-term blood flow restriction increases power output and bar velocity during the bench press. *J. Strength Cond. Res.* 10.1519/JSC.0000000000003649 [Epub ahead of print]. 32379236

[B62] Wilk M. Krzysztofik M. Gepfert M. Poprzecki S. Golas A. Maszczyk A. (2018). Technical and training related aspects of resistance training using blood flow restriction in competitive sport – a review. *J. Hum. Kinet.* 65 249–260. 10.2478/hukin-2018-010100 30687436 PMC6341949

[B63] Wilk M. Krzysztofik M. Jarosz J. Krol P. Leznicka K. Zajac A. (2021b). Impact of ischemic intra-conditioning on power output and bar velocity of the upper limbs. *Front. Physiol.* 12:626915. 10.3389/fphys.2021.626915 33716773 PMC7947627

[B64] Wilk M. Krzysztofik M. Maszczyk A. Chycki J. Zajac A. (2019c). The acute effects of caffeine intake on time under tension and power generated during the bench press movement. *J. Int. Soc. Sports Nutr.* 16 8. 10.1186/s12970-019-0275-x 30777094 PMC6379960

[B65] Yasuda T. Fukumura K. Iida H. Nakajima T. (2015). Effect of low-load resistance exercise with and without blood flow restriction to volitional fatigue on muscle swelling. *Eur. J. Appl. Physiol.* 115 919–926. 10.1007/s00421-014-3073-9 25491331

